# A validation of 11 body-condition indices in a giant snake species that exhibits positive allometry

**DOI:** 10.1371/journal.pone.0180791

**Published:** 2017-07-19

**Authors:** Bryan G. Falk, Ray W. Snow, Robert N. Reed

**Affiliations:** 1 U.S. Geological Survey, Fort Collins Science Center, Fort Collins, Colorado, United States of America; 2 National Park Service, Everglades National Park, Homestead, Florida, United States of America; University of Regina, CANADA

## Abstract

Body condition is a gauge of the energy stores of an animal, and though it has important implications for fitness, survival, competition, and disease, it is difficult to measure directly. Instead, body condition is frequently estimated as a body condition index (BCI) using length and mass measurements. A desirable BCI should accurately reflect true body condition and be unbiased with respect to size (i.e., mean BCI estimates should not change across different length or mass ranges), and choosing the most-appropriate BCI is not straightforward. We evaluated 11 different BCIs in 248 Burmese pythons (*Python bivittatus*), organisms that, like other snakes, exhibit simple body plans well characterized by length and mass. We found that the length-mass relationship in Burmese pythons is positively allometric, where mass increases rapidly with respect to length, and this allowed us to explore the effects of allometry on BCI verification. We employed three alternative measures of ‘true’ body condition: percent fat, scaled fat, and residual fat. The latter two measures mostly accommodated allometry in true body condition, but percent fat did not. Our inferences of the best-performing BCIs depended heavily on our measure of true body condition, with most BCIs falling into one of two groups. The first group contained most BCIs based on ratios, and these were associated with percent fat and body length (i.e., were biased). The second group contained the scaled mass index and most of the BCIs based on linear regressions, and these were associated with both scaled and residual fat but not body length (i.e., were unbiased). Our results show that potential differences in measures of true body condition should be explored in BCI verification studies, particularly in organisms undergoing allometric growth. Furthermore, the caveats of each BCI and similarities to other BCIs are important to consider when determining which BCI is appropriate for any particular taxon.

## Introduction

As a measure of the relative energy stores of an animal [1], body condition has both theoretical and practical importance because of its strong associations with reproductive capacity [2, 3], survivorship [3–5], competition [6, 7], and disease [8–10]. Despite this importance, body condition is generally unfeasible to measure directly because direct measurement requires destruction of the whole specimen [11–14]. Instead, biologists frequently estimate body condition using a body condition index (BCI) calculated from mass and length variables obtained from non-invasive measurements (reviewed in [14]; see [15] for alternative means to estimate body condition).

There are many BCIs based on length and mass, and most can be categorized into two groups: ratio BCIs and regression BCIs (Table 1). The first proposed BCIs were ratio BCIs, and these are still commonly used today (e.g., the body mass index for humans is the Quételet index, or mass/length^2; [16, 17]). Ratio BCIs are intuitive and are comparable among individuals from different statistical populations, but they are plagued with bias (described below; [1, 18]). Regression-based BCIs are also commonly used; the residuals from a regression of log-transformed mass on log-transformed length (i.e., the observed mass minus the expected mass) are the BCI. These BCIs, where a positive value indicates a fat animal and a negative value indicates a skinny animal, are also intuitive, but they must satisfy a number of assumptions, including: the data are linear, the variance is homoscedastic, and the residuals are normally distributed [1, 19]. Furthermore, Type I regressions (e.g., ordinary least squares or OLS regressions, which are commonly employed as BCIs) assume that body length as the independent variable is known without error so that variation around the best-fit line is attributable only to variation in mass as the dependent variable, but there are a number of potential sources of error in body-length data [19]. Also, and unlike the ratio BCIs, estimates from regression BCIs cannot be compared among individuals from different statistical populations because the regression slopes among populations may be different [18, 19]. Not surprisingly, choosing which BCI to use is not straightforward, and different studies have championed different BCIs as most appropriate [13, 18, 20–23].

**Table 1:** Summary of body condition indices (BCIs) used in this study. When available, the names were derived from the literature. Otherwise, we gave arbitrary, descriptive names to unnamed BCIs to facilitate communication.

Description	Abbreviation	Name	Category	Citations
Body mass divided by body length	M/L	Ratio index	Ratio	[14]
Body mass divided by body length squared	M/L^2	Quételet index	Ratio	[17]
Body mass divided by body length cubed	M/L^3	Fulton’s index	Ratio	[24, 25]
Body mass divided by predicted body mass from SMA regression	M/prM	Relative index	Ratio	[26]
Log-transformed body mass divided by log-transformed body length	logM/logL	Log ratio index	Ratio	[14]
Log-transformed body mass divided by log-transformed predicted body mass from SMA regression	logM/log(prM)	Log relative index	Ratio	[1]
Residuals from OLS linear regression of log-transformed body mass on log-transformed body length	OLSres	OLS residual index	Regression (Type I)	[18]
Residuals from MA linear regression of log-transformed body mass on log-transformed body length	MAres	MA residual index	Regression (Type II)	[13, 19]
Residuals from SMA linear regression of log-transformed body mass on log-transformed body length	SMAres	SMA residual index	Regression (Type II)	[13, 19]
Residuals from SMA linear regression of body mass on body length cubed	res(M~L^3)	Cubed regression index	Regression (Type II)	[22]
Scaled Mass Index	SMI	Scaled mass index (SMI)	Allometric	[27]

OLS = Ordinary least squares

MA = Major axis

SMA = Standardized major axis

An ideal BCI should be accurate (i.e., correlated with true body condition) and unbiased with respect to size (i.e., not correlated with body mass or a linear length measurement; [14]). The requirement that the BCI is accurate is straightforward because body condition is what the BCI is estimating. The requirement that the BCI is unbiased with respect to size is important for hypothesis testing, because a lack of correlation with size allows a researcher to compare BCI estimates across individuals of different size ranges [11, 14]. Generally speaking, ratio BCIs are correlated with size but by definition residual BCIs are not [1, 11].

Many animal species undergo allometric rather than isometric growth, meaning that their shape changes over their lifespan as their mass increases or decreases in proportion to length, and allometric changes are another potential challenge to BCI inference [19, 28]. One BCI was specifically proposed as a solution to the allometry problem–the scaled mass index (SMI; [27]). The SMI accommodates allometric changes using the Thorpe-Lleonart (TL) scaling model:

$$Y_{i}^{*}=Y_{i}{\left[\frac{X_{0}}{X_{i}}\right]}^{b_{SMA}}$$ (1)

where $Y_{i}^{*}$ is the predicted value of Y (mass) for individual i after correcting for the scaling relationship between X (length) and Y (mass); X_i_ and Y_i_ are the observed values of X (length) and Y (mass) for individual i; X_0_ is the arithmetic mean of X (length) for the study population (this value is arbitrary and can be any value of X observed in the study population); and b_SMA_ is the slope of a standardized (reduced) major axis (SMA) regression of log-transformed mass on log-transformed length for the study population [27, 29]. This approach eliminates all allometric effects on size [29], has few assumptions, and is comparable among populations, but how well the SMI correlates with true body condition is variable among datasets [20–22].

Here we explore the performance of BCIs in a large constricting snake species, the Burmese python (*Python bivittatus*). Snakes are perhaps ideal animals to explore BCI performance because their body plan is simple; they do not have appendages or other additional features that may change in shape and confound BCI inference. Like other squamates, pythons store fat in discrete fat bodies in their coelomic cavity, and the fat bodies can be removed and weighed during necropsy (i.e., wet-fat mass; [30]). In viperid and colubrid snakes, both the wet and dry weights for whole bodies and the wet and dry weights for fat bodies are highly correlated, suggesting that the proportional mass of water, organic matter, and inorganic matter in both whole bodies and fat bodies in snakes is constant [31].

This consistency in wet and dry weights of fat mass makes wet-fat mass available to use in a measure of ‘true’ body condition to evaluate BCIs in snakes and likely other squamates. Wet-fat mass has been used in a measure of true body condition to evaluate BCIs [32, 33] and body condition scores (i.e., numerical values that are associated with specific body-condition categories [e.g., shoulders are ‘v-shaped’]; [33, 34]) in squamates and in other taxa. Nonetheless, there are factors that may affect how well measures using wet-fat mass approximate true body condition. For example, fat may be stored elsewhere in addition to the fat bodies (e.g., the liver; [32]). Also, other tissues–most notably muscle–contribute to body condition [14], and fat mass ignores the contribution of these other tissues. These caveats likely reduce the accuracy of wet-fat mass as an approximation of true body condition, but we posit that BCI evaluation using such an approximation is justifiable in an animal species that is otherwise too large to evaluate by traditional methods (i.e., drying and grinding the specimen prior to lipid extraction; [11]).

Dry- and wet-fat mass have been used in a variety of ways to approximate true body condition and verify BCIs. Common measures include both total fat mass and percent fat (total fat mass / total body mass; [11, 13, 18, 22, 27, 35]). Fat mass and percent fat do not accommodate allometric changes in true body condition; if an animal exhibits allometry in true body condition, then these measures will be biased with respect to animal size. Measures of true body condition that accommodate allometry include scaled fat, where fat mass is scaled to length using the TL scaling model described above [27], and residual fat, where fat mass is regressed on length to calculate residuals [22]. Neither scaled fat nor residual fat are expected to change with body size [29, 36].

We identified 11 BCIs from the literature (Table 1) and assessed their performance using wet-fat masses of Burmese pythons collected in Florida as part of invasive-species removal efforts. We characterized the allometric relationship between mass and length for these snakes, considered percent fat, scaled fat, and residual fat as alternative measures of true body condition, tested these for an association with snake length (i.e., allometry), and used them to assess BCI accuracy.

## Methods

We performed necropsies on Burmese pythons that were collected in southern Florida during 2004–2014 as part of ongoing invasive-species management activities. The snakes were humanely euthanized via captive bolt, kept on ice, and either necropsied within 24 hours of euthanasia or frozen and later thawed on ice for necropsy. No Institutional Animal Care and Use Committee approval was necessary because the invasive pythons were euthanized as part of management–and not research–activities, but methods of safe and humane euthanasia were developed in consultation with the National Park Service Wildlife Health Team. Similarly, these necropsies were not performed explicitly to evaluate BCIs, but we nonetheless collected the necessary data. We used measurements of total body mass (g) and snout-vent length (SVL; cm) as body-mass and length metrics for our BCIs. We used SVL instead of total length because SVL is more tightly associated with mass than is total length in snakes (because tail length varies; [37]). During necropsy we removed all visible fat from the coelomic cavity and weighed it (wet-fat mass; g). We recorded sex (male or female) and for a subset of individuals we recorded whether the snake was necropsied while fresh or frozen and later thawed. We excluded pythons that were not immediately put on ice or frozen after death (e.g., we excluded pythons found dead on roads). We performed all subsequent analyses in R v.3.3.3 [38] using the car [39], corrplot [40], cowplot [41], extrafont [42], fBasics [43], ggplot2 [44], lmodel2 [45], lmtest [46], moments [47], and smatr [48] packages.

We wanted to know whether differences in sex or in our specimen-handling procedures (i.e., storing on ice vs. freezing/thawing prior to necropsy) are associated with differences in the relationship between wet-fat mass and SVL in our dataset because if they are, these groups should be treated separately in downstream analyses. We used likelihood ratio tests for common slopes [49] to test the null hypotheses that the slopes of SMA regressions of log-transformed wet-fat mass on log-transformed SVL are equal between sexes and between frozen and fresh specimens. We had complete specimen-handling information for 73 specimens, so we constrained the specimen-handling test to this smaller dataset. We consider p ≤ 0.05 for these and similar tests to be statistically significant but acknowledge that applying a p-value threshold for significance is arbitrary [50].

We calculated percent fat, scaled fat, and residual fat for each snake as alternative measures of true body condition. Percent fat was wet-fat mass divided by total body mass. We calculated scaled fat using measurements of wet-fat mass and SVL and the TL scaling model. We calculated residual fat by taking the residuals from a SMA regression of log-transformed wet-fat mass on log-transformed SVL. Because we observed an influence of sex on the relationship between wet-fat mass and SVL (see Results), we calculated scaled fat and residual fat for each of these groups separately and conducted all downstream analyses separately by sex.

We characterized the relationship between body length and body mass, fat mass, percent fat, scaled fat, and residual fat. We used a SMA regression of log-transformed mass on log-transformed SVL and of log-transformed fat mass on log transformed SVL to infer the regression slopes (i.e., the slopes of the allometric lines for total mass and fat mass, respectively) and used likelihood ratio tests to test the null hypotheses that the slopes between sexes are equal. An ideal measure of true body condition is not associated with body length, so we estimated the slopes and r^2^ values from OLS regressions of percent fat, scaled fat, and residual fat on log-transformed SVL; if there is no relationship between the body-condition measure and body length, then the r^2^ value will be zero and the slope will be non-significant. We used OLS regressions to characterize the relationship between the true body condition measures and length because in this context any variation around the best-fit line is attributable only to variation in the dependent variable as the values for length in both the dependent and independent variables are equal.

We calculated each of the 11 BCIs (Table 1) for each python. The residual BCIs have three basic assumptions: 1) the data are linear; 2) the variance is constant (i.e., homoscedastic); and 3) the frequency distribution of residuals is normal. We tested for linearity using Ramsey’s RESET test [51] and for non-constant variance using the Breusch-Pagan test [52]. We tested for normality using the Shapiro-Wilk test [53]. For the residual BCIs for which we rejected normality, we tested for skewedness using the D'Agostino test [54] and for kurtosis using the Anscombe-Glynn test [55].

An optimal BCI closely approximates true body condition and is unbiased with respect to body size, and we characterized this relationship in several ways. First, we estimated the Kendall rank correlation coefficient (τ; [56]) to test for a correlation between each BCI and percent fat, scaled fat, and residual fat as measures of true body condition (i.e., accuracy) and SVL as a measure of size (i.e., bias). Second, and similarly, we regressed each BCI against percent fat, scaled fat, residual fat, and log-transformed SVL for each sex and estimated the proportion variation in each of the response variables that can be explained by SVL (r^2^).

We wanted to know to what extent any given BCI could be substituted for another, and so we created a correlation matrix to explore how similar each of the BCIs are to each other with our dataset. For each sex separately, we generated pairwise Kendall rank correlation coefficients between inferred BCI values.

The BCIs most strongly correlated with percent fat were biased with respect to length (see Results), so we explored the extent of the bias. More specifically, we wanted to know whether these BCIs exhibited size bias among pythons that differ in SVL by ≤ 0.5 m in our dataset. We partitioned both female and male datasets into two subsets: pythons ≤ 0.25 m of the mean SVL and pythons ≥ 0.25 m of the mean SVL. For each sex and using the BCIs identified as best-fitting by r^2^ values as described above, we used Mann-Whitney U tests [57] to test the hypothesis that BCI values from each of the two size categories are pulled from the same distribution. A non-significant p-value for these tests would suggest that BCI estimates of pythons that differ by ≤ 0.5 m SVL in our dataset are comparable. SVL measurements for Burmese pythons collected in southern Florida range approximately 0.5–5.0 m for females and 0.5–3.5 m for males (Table 2), and we believe that 0.5 m is the smallest possible SVL size category that may allow sufficient sample sizes for future hypothesis testing (e.g., are Burmese pythons getting skinnier because of a lack of available prey?).

**Table 2:** Summary of snout-vent length (SVL), total mass, wet-fat mass, percent fat, scaled fat, and residual fat data from 248 Burmese pythons collected in southern Florida during 2004–2014. Values are expressed as: mean (total range). Females in this dataset are generally longer, heavier, and fatter than males.

Sex	N	SVL (cm)	Total Mass (g)	Wet-fat Mass (g)	Percent Fat	Scaled Fat	Residual Fat
Female	114	235 (70–482)	11,852 (186–75,500)	1226 (5.0–8406)	0.0824 (0.00652–0.173)	772 (55.2–3076)	0.0 (-2.43–1.59)
Male	134	214 (76–347)	7683 (260–32,600)	571 (4.0–4374)	0.0645 (0.00827–0.143)	451 (38.8–1023)	0.0 (-2.32–0.943)

## Results

We obtained records of total mass, wet-fat mass, SVL, and sex for 248 Burmese pythons that were immediately placed on ice or frozen after death. Of these, 137 were male and 109 were female, and males were usually smaller and had less fat than females (Table 2). We rejected the null hypothesis that differences in sex do not affect the relationship between wet-fat mass and SVL (p ≤ 0.05), and so we separated males and females in all downstream analyses. In contrast, we could not reject the null hypothesis that our specimen-handling procedures (i.e., whether the animals had been frozen prior to necropsy) do not affect the relationship between wet-fat mass and SVL (p = 0.28), so we ignored specimen-handling information in downstream analyses.

**Fig 1:**
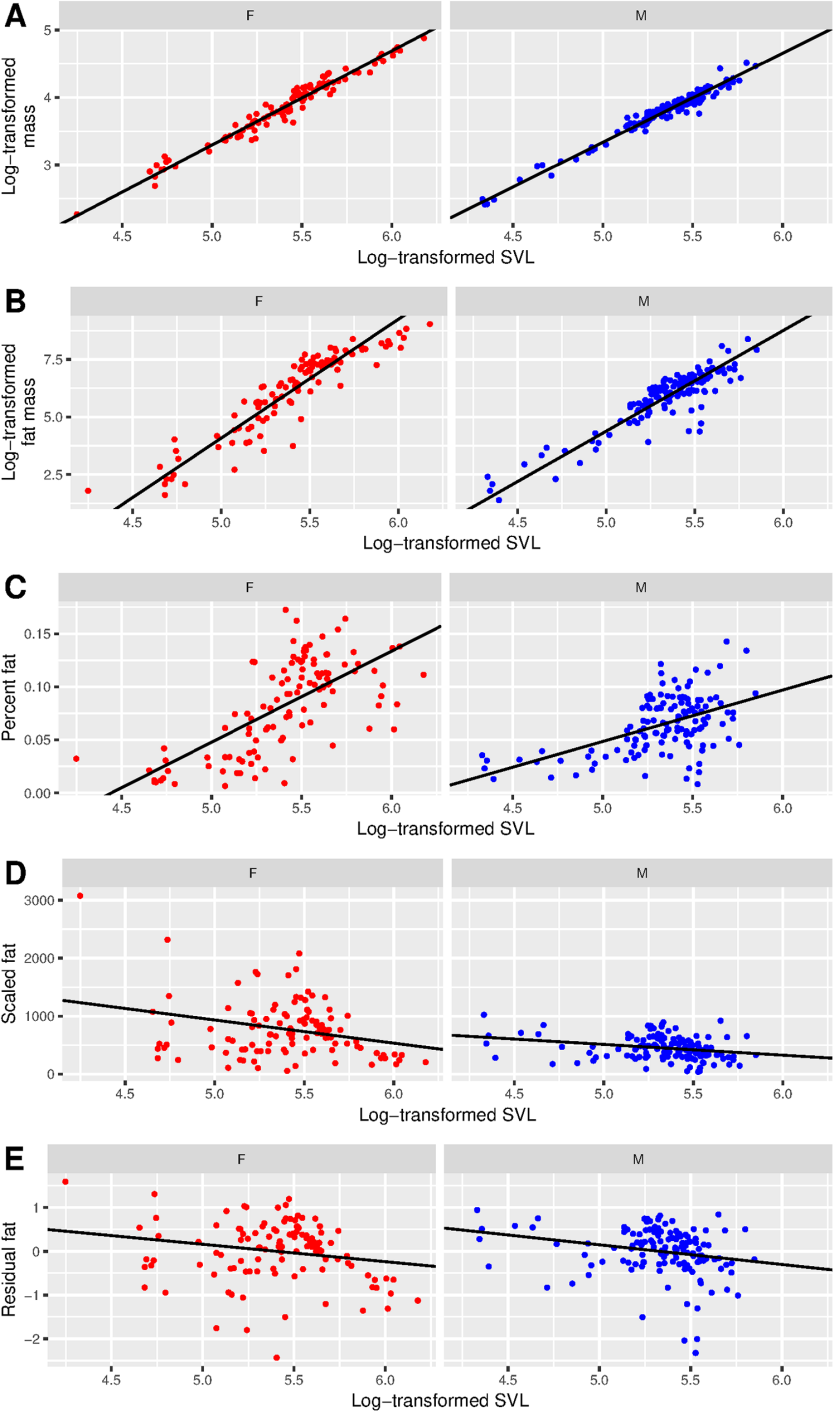
Relationships between snout-vent length (SVL) and mass, fat, and true body condition measures for male and female Burmese pythons. The SMA regression of log-transformed body mass on log-transformed snout-vent length (A) and of log-transformed fat mass on log-transformed SVL (B) shows the allometric relationship among these variables. As measures of true body condition, OLS regressions of percent fat on log-transformed SVL (C), scaled fat on log-transformed SVL (D), and residual fat on log-transformed SVL (E) should have zero slopes, but they do not. Significance for the regression slopes and r^2^ values can be found in Table 3.

Females exhibited more positive allometry than males in both total mass and fat mass, and fat mass was more positively allometric than total mass in both sexes (Table 3, Fig 1A and 1B). The slope of the SMA regression (i.e., the allometric line) for total mass was 3.21 [95% CI: 3.10–3.33] for females and 3.04 [95% CI: 2.94–3.14] for males (Fig 1A). The 95% CIs for these slope estimates slightly overlap between the two sexes, but we rejected the hypothesis that the slopes are equal (p ≤ 0.05; i.e., male and female pythons exhibit different allometric relationships between length and total mass). The slope of the SMA regression for fat mass was 5.16 [95% CI: 4.81–5.55] for females and 4.37 [95% CI: 4.06–4.72] for males (Fig 1B). We also rejected the hypothesis that these slopes are equal (p ≤ 0.05; i.e., male and female pythons also exhibit different allometric relationships between length and fat mass; Table 3).

**Table 3:** Relationships between body length and mass, fat, and true body condition measures for male and female Burmese pythons. We estimated the probability that the slope is not zero (i.e., p ≤ 0.05) and calculated the r^2^ values from SMA regressions of log-transformed body mass and log-transformed wet-fat mass on log-transformed snout-vent length (SVL) and from OLS regressions of percent fat, scaled fat, and residual fat on log-transformed SVL. Ideally, measures of true body condition will have no relationship with body length (i.e., percent fat, scaled fat, and residual fat should have non-significant regression slopes and r^2^ values of zero), but each of them do. These results are visualized in Fig 1.

	Female		Male	
Variable	Slope	r^2^	Slope	r^2^
Body mass	**	0.96	**	0.96
Fat mass	**	0.85	**	0.81
Percent fat	**	0.45	**	0.26
Scaled fat	*	0.078	*	0.068
Residual fat	*	0.038	*	0.051

* p ≤ 0.05

**p ≤ 0.001

Each of our measures of true body condition was associated with size (Table 3; Fig 1C–1E). Percent fat exhibited the strongest relationship with SVL, having a significant positive slope (p ≤ 0.001) and r^2^ values that suggest > 40% of the variation in percent fat is explained by SVL. Scaled fat exhibited a slight but significant negative relationship with SVL (p ≤ 0.05), and the r^2^ values suggest that approximately 7% of the variation in scaled fat is explained by SVL. Residual fat was similar, exhibiting a slightly negative but still significant relationship with SVL (p ≤ 0.05), and the r^2^ values suggest that 4–5% of the variation in residual fat is explained by SVL.

The testable assumptions of the regression-based BCIs were satisfactorily met in many cases, but not all (Table 4). We could not reject linearity or homoscedasticity for regressions of log-transformed mass on log-transformed length, and though we rejected normality for these regressions in females, we could not reject deviations due skewedness or kurtosis, suggesting that deviations from normality are minimal (i.e., assumptions for the OLS, MA, and SMA regressions were generally met in both sexes). Though we could not reject linearity, we strongly rejected both homoscedasticity and normality for the regression of mass on length cubed.

**Table 4:** Tests of assumptions of the regression-based body condition indices (BCIs). We used several tests to test the hypotheses that: 1) the data are linear; 2) the variance is constant (i.e., homoscedastic); and 3) the frequency distribution of residuals is normal. For clarity, only p-values are reported. Most assumptions are met for regressions of log-transformed mass on log-transformed length, but regressions of mass on length cubed exhibited neither homoscedasticity nor normality. Abbreviations for each of the BCIs are provided in Table 1.

BCI	Regression	Ramsey’s RESET Test (linearity)		Breusch-Pagan Test (homoscedasticity)		Shapiro-Wilk Test (normality)		d’Agostino Test (skewness)		Anscombe-Glynn Test (kurtosis)	
		F	M	F	M	F	M	F	M	F	M
OLSres	OLS (logM~logL)	—	—	—	—	*	—	—	n/a	—	n/a
MAres	MA (logM~logL)					*	—	—	n/a	—	n/a
SMAres	SMA (logM~logL)					*	—	—	n/a	—	n/a
res(M~L^3)	SMA (M~L^3)	—	—	**	**	**	**	**	**	**	**

— p > 0.05

* p ≤ 0.05

**p ≤ 0.001

**Fig 2:**
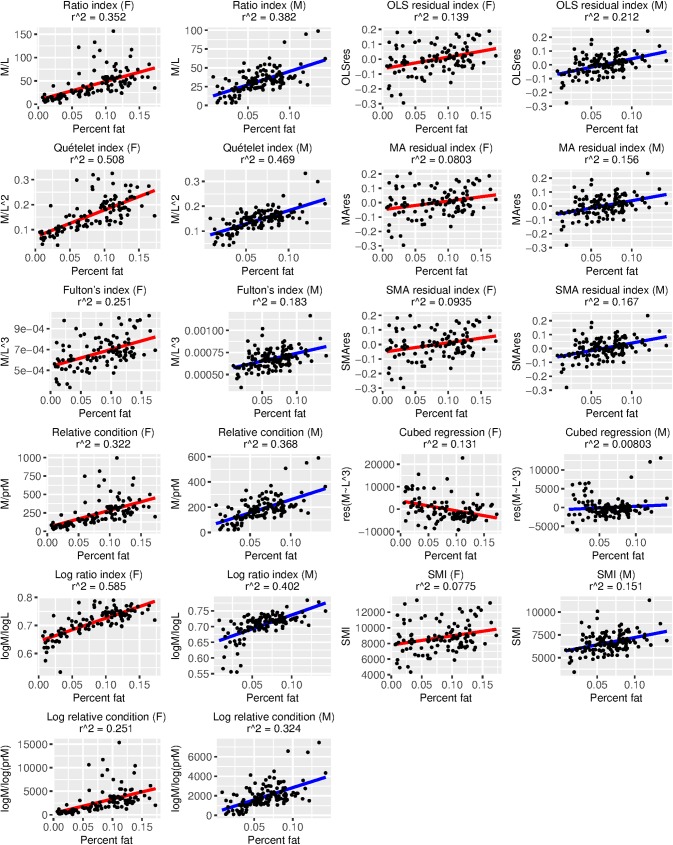
Relationships between 11 body condition indices (BCIs) and percent fat, a measure of true body condition, for each sex. Variation in each BCI that can be explained by percent fat is provided as an r^2^ value. Ideally, most variation in a BCI should be attributable to variation in true body condition, and there should be a positive linear relationship between the BCI and true body condition. Descriptions of each BCI are provided in Table 1.

**Fig 3:**
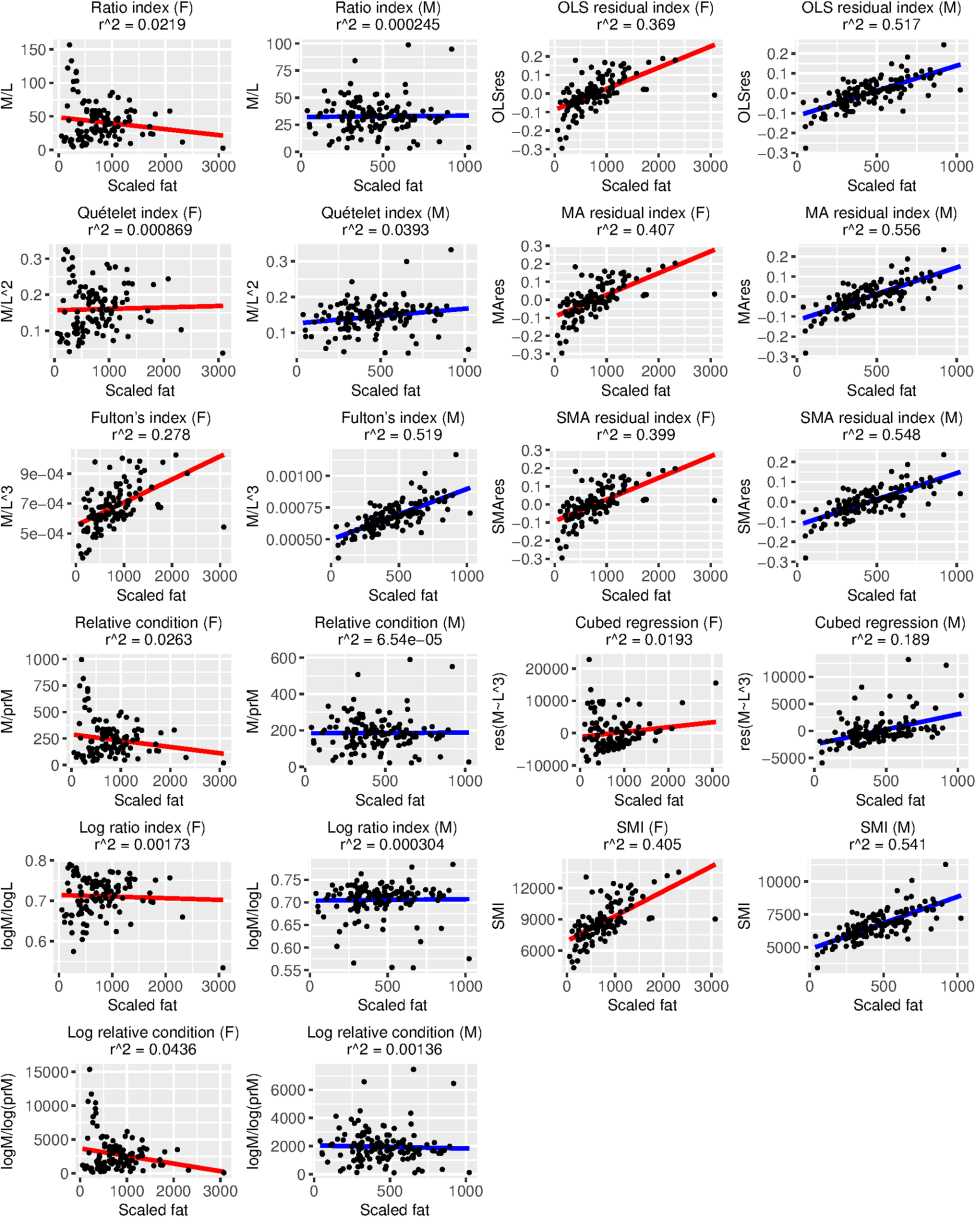
Relationships between 11 body condition indices (BCIs) and scaled fat, a measure of true body condition, for each sex. Variation in each BCI that can be explained by scaled fat is provided as an r^2^ value. Ideally, most variation in a BCI should be attributable to variation in true body condition, and there should be a positive linear relationship between the BCI and true body condition. Descriptions of each BCI are provided in Table 1.

**Fig 4:**
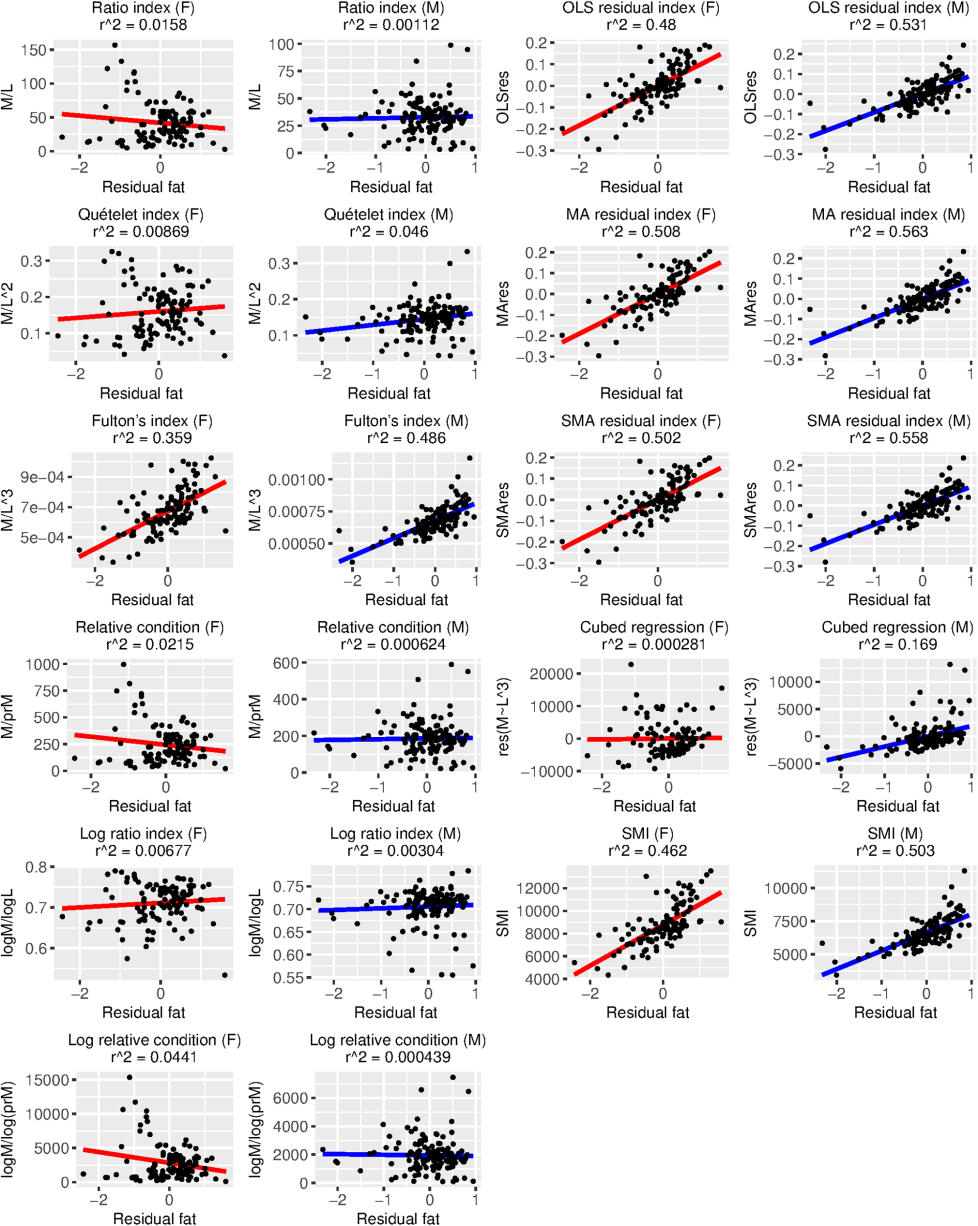
Relationships between 11 body condition indices (BCIs) and residual fat, a measure of true body condition, for each sex. Variation in each BCI that can be explained by residual fat is provided as an r^2^ value. Ideally, most variation in a BCI should be attributable to variation in true body condition, and there should be a positive linear relationship between the BCI and true body condition. Descriptions of each BCI are provided in Table 1.

**Fig 5:**
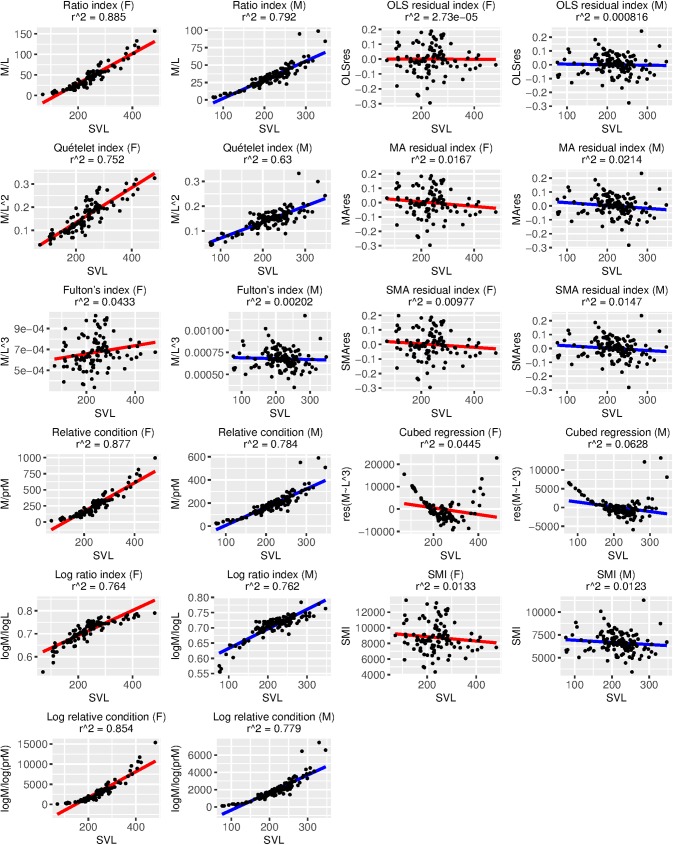
Relationships between 11 body condition indices (BCIs) and snout-vent length (SVL) for each sex. Variation in each BCI that can be explained by SVL is provided as an r^2^ value. An ideal BCI has no relationship (i.e., zero regression slope and zero r^2^ value) with SVL. Descriptions of each BCI are provided in Table 1.

BCI performance was variable and depended heavily on which measure of true body condition we employed (Table 5, Figs 2–5). Using percent fat as a measure of true body condition, the log ratio index in females (τ = 0.56, Table 5; r^2^ = 0.59, Fig 2) and the Quételet index in males (τ = 0.46, Table 5; r^2^ = 0.47, Fig 2) were among the best-performing, but each of these was more strongly associated with SVL than percent fat (log ratio index in females: τ = 0.76, Table 5; r^2^ = 0.76, Fig 5; Quételet index in males: τ = 0.76, Table 5; r^2^ = 0.55, Fig 5). The best-performing BCIs using scaled fat and residual fat as measures of true body condition contrasted with the results using percent fat. For example, the MA residual index had the highest r^2^ values (0.41–0.56; Figs 3 and 4) and τ values (0.54–0.56; Table 5) in both sexes for scaled fat and residual fat. Notably, only ~2% of the variation in the MA residual index is associated with SVL (Fig 5), but though this relationship is small, it is significant in males (τ = -0.14, p ≤ 0.05; Table 5).

**Table 5:** Kendall’s τ correlation coefficients between body condition indices (BCIs) and percent fat, scaled fat, residual fat, and snout-vent length (SVL). A desirable BCI is strongly correlated with ‘true’ body condition (i.e., percent fat, scaled fat, or residual fat) but is not correlated with size (i.e., SVL). Values with the greatest correlation coefficient for each column are italicized. Generally speaking, BCIs that are strongly correlated with percent fat are also strongly correlated with SVL in our dataset. Abbreviations for each of the BCIs are provided in Table 1.

BCI	Percent fat		Scaled fat		Residual fat		SVL	
	F	M	F	M	F	M	F	M
M/L	0.56**	0.41**	0.03	-0.02	0.03	-0.02	0.83**	0.77**
M/L^2	*0*.57**	*0*.46**	0.11	0.15*	0.11	0.15*	0.71**	0.55**
M/L^3	0.35**	0.29**	0.45**	0.54**	0.45**	0.54**	0.21*	-0.09
M/prM	0.56**	0.41*	0.03	-0.02	0.03	-0.02	0.83**	0.78**
logM/logL	0.56**	0.44**	0.07	0.08	0.07	0.08	0.76**	0.65**
logM/log(prM)	0.54**	0.39*	0.00	-0.06	0.00	-0.06	*0*.86**	*0*.82**
OLSres	0.25**	0.30**	0.52**	0.54**	0.52**	0.54**	0.03	-0.07
MAres	0.19*	0.25**	*0*.54**	*0*.56**	*0*.54**	*0*.56**	-0.06	-0.1*
SMAres	0.20*	0.26**	*0*.54**	0.55**	*0*.54**	0.55**	-0.04	-0.13*
res(M~L^3)	-0.24**	0.01	0.08	0.33**	0.08	0.32**	-0.40**	-0.28**
SMI	0.20*	0.26**	*0*.54**	0.55**	*0*.54**	0.55**	-0.04	-0.13*

* p≤0.05

**p≤0.001

**Fig 6:**
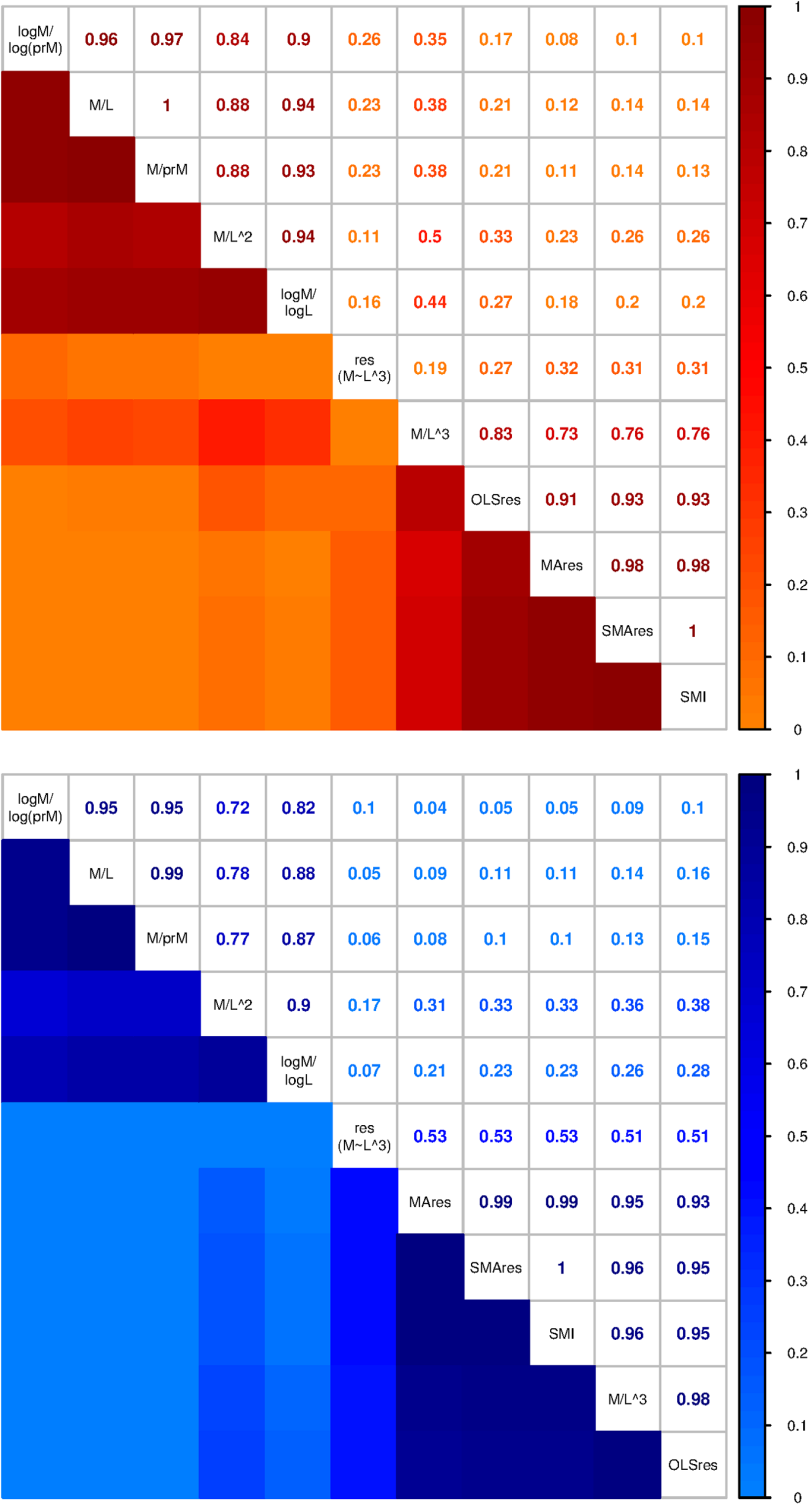
Correlations (Kendall’s τ) among 11 body condition indices (BCIs) for each sex. Most BCIs fall into two groups, and certain BCIs are very similar to others (e.g., the MA residual index, the SMA residual index, and the SMI are all strongly correlated to each other). Descriptions of each BCI are provided in Table 1.

The correlations among BCIs revealed that most BCIs fall out into one of two groups, where a group is characterized by pairwise Kendall rank correlation coefficients of τ > 0.7 (Fig 6). One group contains all the ratio indices except the Fulton index. The other group contains the Fulton index, the SMI, and all the regression indices except the cubed regression. Notably, the MA residual index, SMA residual index, and the SMI were very strongly correlated to each other in both sexes (τ ≥ 0.98). The cubed regression was dissimilar to all others (τ = 0.05–0.53).

We tested the extent of the length bias for the log ratio index for females and the Quételet index for males because these had the highest r^2^ values in a regression on percent fat (Fig 2). After creating a length category ≥ 0.25 m and another ≤ 0.25 m than the mean SVL for each sex, we rejected the hypothesis that the two length categories exhibit the same BCIs in females (W = 43, p ≤ 0.05) but not males (W = 333, p = 0.14). In other words, the log ratio index for females exhibits significant length bias in 0.5 m length categories in our dataset, but the Quételet index for males does not.

## Discussion

We evaluated 11 BCIs using percent fat, scaled fat, and residual fat as alternative measures of true body condition in Burmese pythons. In our dataset, females exhibit a more positive allometric relationship between mass and length and between fat mass and length than do males, and fat mass is strongly associated with length in both sexes. None of our three measures of true body condition completely removed this effect of size, though the effects of size were minor for scaled fat and residual fat. Our inferences of the best-performing BCIs heavily depended on our measure of true body condition. BCIs exhibiting strong associations with percent fat often exhibited even stronger relationships with SVL. These BCIs included most of the ratio indices (ratio index, Quételet index, relative condition index, log ratio index, and log relative condition index). Conversely, BCIs with strong associations with scaled fat and residual fat exhibited weak or no relationships with SVL. These BCIs included most of the regression indices (OLS residual index, MA residual index, and SMA residual index), the Fulton index, and the SMI.

It is unclear to what extent BCI evaluation in other taxa may be sensitive to the chosen measure of true body condition. In both sexes of house mice, for example, the best-performing BCI was the same when evaluated against both percent fat and residual fat (that best-performing BCI was a multiple regression containing skeletal measurements and is not otherwise comparable to our study; [22]). In an evaluation of the OLS residual index in several species (e.g., chipmunks, meadow voles, starlings, and watersnakes), the correlations with percent fat and with scaled fat were sometimes similar and sometimes different [27]. In chipmunks, for example, the coefficient dropped by more than half from 0.3 (scaled fat) to 0.12 (percent fat), but in watersnakes it remained relatively stable increasing slightly from 0.49 (scaled fat) to 0.54 (percent fat; [27]). This latter result is particularly interesting in the context of our study because it is also a snake with a simple body plan, but that species exhibits an opposite trend in association between the OLS residual index and percent fat and between the OLS residual index and scaled fat because in Burmese pythons, the OLS residual index is more strongly associated with scaled fat than percent fat (Table 5). This difference may be a result of differences in allometry between Burmese pythons and watersnakes. We are not aware of any other studies that compared BCI performance when using percent fat, scaled fat, residual fat, or other transformations of fat mass as measures of true body condition, and additional work is necessary to demonstrate whether BCI evaluation in other taxa is sensitive to interpretation of true body condition.

We consider scaled fat and residual fat to be preferable approximations of true body condition because these measures exhibit minimal association with length. Though minimal, they were unfortunately not free from a size bias. The source of the bias may be an artefact of our dataset, as the residuals from the SMA regression of log-transformed fat mass on log-transformed SVL (these residuals are residual fat, a measure of true body condition, and this regression slope is included in the TL scaling model to calculate scaled fat, which is another measure of true body condition) are leptokurtic in males (6.17, p ≤ 0.001) and negatively skewed in both females (-0.71, p ≤ 0.05) and males (-1.4, p ≤ 0.001). Burmese pythons, particularly males, seem to have a more restrictive upper than lower limit on true body condition (i.e., pythons can be more excessively skinny than excessively fat), and this deviance from a normal distribution is somewhat problematic for calculations of both scaled fat and residual fat because it violates an assumption of the regression analysis.

We consider the SMI, the MA residual index, and the SMA residual index to be the best-performing BCI’s for our dataset because they exhibited a strong association with both scaled fat and residual fat but not SVL. It is difficult to compare BCI performance in Burmese pythons to other taxa because most studies used either fat mass or percent fat (when using fat and not protein, etc., for validation) as a measure of true body condition. Nevertheless, there are some BCI verification data that use scaled fat, and the associations between BCIs and true body condition are widely variable among taxa, so it is no surprise that our results fall within the set of these previous observations. For example, we observed correlation coefficients of 0.54–0.55 between the SMI and scaled fat, and these were as low as -0.151 in deer mice and 0.164 in meadow voles and as high as 0.758 in watersnakes and 0.841 in starlings [27]. The r^2^ values for residual fat and the optimal BCI in house mice (the aforementioned multiple regressions of skeletal measurements that are not comparable to our study) were 0.39 in females and 0.19 in males, whereas we observed r^2^ values for residual fat and the MA residual index of 0.41 in females and 0.56 in males (Fig 4). We found the log ratio index, the Quételet index, and similar BCIs to have size biases that render them inappropriate for use in pythons.

The strong correlations within groups of BCIs are encouraging, because this suggests that–at least in some cases–certain BCIs are interchangeable with others. The SMI, MA residual index, and SMA residual index provided nearly equivalent results in our dataset, as did the ratio index, the relative index, and the log-relative index (Fig 6). The estimated r^2^ values of regressions of the OLS residual BCI on the SMI for five rodent species, starlings, and watersnake ranged 0.636–0.963, with the highest r^2^ value observed in watersnakes [27]. Snakes, with their simple body plans, may exhibit higher correlations among BCIs than other species with legs and wings, and further study is necessary to parse which factors contribute to the strength of correlations among different BCIs.

Though we are not the first to use wet-fat mass to evaluate BCIs (e.g., [3, 32, 33]), it is not common, and some associated limitations warrant discussion. First, fat may be stored elsewhere than in the fat bodies (e.g., the liver; [32]), and our measure of fat mass may be a biased approximation of actual fat mass if accumulation in these other areas does not consistently vary with fat accumulation in the fat bodies. Second, we ignore the contribution of other tissue types to body condition, particularly muscle. Muscle (i.e., protein) is an important component of body condition [14], and the proportions of muscle and fat in Burmese pythons may not covary. Nonetheless, we believe that fat mass is a meaningful biological indicator of body condition in Burmese pythons. Similar to observations in other snakes [58–61], fat may directly influence fitness in Burmese pythons: adults have large fat stores just prior to the breeding season, and these fat stores facilitate a drop in feeding rates as the animals enter their breeding season and actively search for mates (when we observe a Burmese python in Florida without prey remains in its gastrointestinal tract, it is almost always a reproductively capable adult during the breeding season; Falk unpublished). After breeding, females lay eggs and stay with them until they hatch, and the total duration of the reproductive season is enough to deplete the fat stores of these brooding females [62]. Thus, large fat stores may allow more opportunities for both sexes to find mates and may facilitate greater clutch success for females. Note that this additional need for energy reserves by females (i.e., laying and brooding a clutch) is a potential explanation for the higher proportions of fat and more positive length/mass allometry in females vs. males (Table 2; Fig 1).

This reproduction-related caveat brings another potential limitation to using fat mass to evaluate BCIs in Burmese pythons: the total mass of tissues besides fat (and other tissues commonly associated with body condition) in individual Burmese pythons may change according to reproductive and feeding cycles, which in turn may affect the observed proportion of fat. The size and mass of reproductive organs change throughout the year in adult Burmese pythons (Falk unpublished), as do the size and mass of digestion-related organs of all life stages during periods of fasting and eating [63]. This variation affects both dry- and wet-fat mass measurements, and it may introduce noise into our dataset as the proportional mass of these tissues change inversely with the proportion of fat (e.g., if the masses of the follicles and oviducts increase while the remaining tissues remain the same, the proportion of fat mass relative to total mass will decrease), but it is variation and not an introduction of a systematic bias that would allow us to reject our conclusions. Furthermore, these effects as related to digestion may be small; only approximately 10% of Burmese pythons necropsied in Florida have prey in their stomach, when the increase in organ mass may be greatest, and the majority of the digested material is only feathers and hair (i.e., the added mass from feeding and digestion is probably small; [64]).

Finally, we do not know for certain that the proportions of water, organic matter, and inorganic matter remain constant as both whole-body and fat-body masses change in Burmese pythons. Consistency in dry and wet masses has been demonstrated in the other snake species tested [31], and though the pattern has not yet been evaluated in Burmese pythons, there is no evidence to suggest that Burmese pythons would not be the same. To reiterate, we are confident that wet fat is a satisfactory means to approximate true body condition in the context of BCI verification with our dataset, but additional studies that incorporate the dried weights of fat and other tissues in Burmese pythons would be an improvement.

Given the low number of BCI-verification studies relative to the number of studies that use un-verified BCIs to answer ecological questions, employing wet-fat mass as we have here may improve body-condition inference generally. Traditional methods of BCI verification involve drying the specimen in an oven, grinding it, and then extracting and quantifying each of the remaining components (e.g., fat, organic matter, inorganic matter; [14]). While feasible for a limited number of small-sized animals, this approach becomes much less practical for larger animals. This association between body size and feasibility has resulted in a bias in the BCI-verification literature towards small-bodied subjects including arthropods, rodents, songbirds, etc. (e.g., [13, 18, 20–22]). Incorporating alternative approaches to BCI evaluation, including using wet-fat mass as we did here, may result in a larger number of species for which we have verified BCIs, which in turn may result in a better understanding of how BCIs can be appropriately applied to ecological questions.
